# Bibliograpical

**Published:** 1896-10

**Authors:** 


### Bibliographical.

Dental Chemistry and Metallurgy. Fourth Edition, Revised and Enlarged, with many Illustrations. By Clifford Mitchell, A. M., M.D.

The fourth edition of this work has recently been issued, largely revised, much enlarged, and made better adapted to the wants of the dental student for whose needs it has been more especially prepared. More than one hundred pages have been added to the book, containing matter entirely of a practical kind that serves to illustrate the principles covered by the five preceding chapters. This new work upon Experimental Chemistry has been followed by an outline on Chemical Analysis, the recognition of the more usual metals being considered at much greater length than in previous editions. Experimental work in this edition includes Physiological Chemistry, in which that of digestion is treated at considerable length. All the matter pertaining to Dental Chemistry has been retained and considerably more added. The work is a most admirable one for use in dental colleges and should find a place in every dental college library; indeed, every dental student should possess a copy, and not only possess it, but should take it in, digest it, and make it a part of himself.

The various subjects presented are treated very clearly, concisely, and not overloaded with verbiage. Indeed, they are remarkably condensed, considering that the ideas are so fully presented. It is impossible, in a few words here, to give an idea of the real value of the work; *that* can only be attained by a full and careful study of the book itself. While this treatise is invaluable to the dental student, it is quite as valuable to the dental practitioner who desires to inform himself on these subjects and keep abreast with the times, so we have no hesitancy in saying to every member of the profession, as well as to every dental student, let this book find a place in your library, and let it receive the consideration it merits at your hands. The work is published by the W. T. Keener Company, of Chicago. Its mechanical execution is excellent, with splendid type, excellent paper, and is put up in very substantial, neat form. It can be obtained from the publishers or through any book-seller.

